# Green and Efficient Determination of Iron(III) in Gasoline Using Microemulsion-Based Extraction and Digital Image Analysis

**DOI:** 10.1021/acsomega.5c08435

**Published:** 2025-12-03

**Authors:** Jordana de Assis Nunes Oliveira, James Michael Silva, Eduarda Garcia Santana, Wallace Henrique Cardoso Peres, Weida Rodrigues Silva, João Flávio da Silveira Petruci, Vanessa Nunes Alves

**Affiliations:** † 583274 Federal University of Catalão (UFCAT), Institute of Chemistry, Av. Dr. Lamartine Pinto de Avelar, 1120, Catalão, Goiás 75704-020, Brazil; ‡ Federal University of Uberlãndia (UFU), Institute of Chemistry, Av. João Naves de Ávila 2121, Uberlândia, Minas Gerais 38408-100, Brazil

## Abstract

Iron­(III) contamination in gasoline, originating from production or storage, compromises fuel quality and engine performance by catalyzing oxidation reactions, promoting gum and sediment formation, and accelerating engine wear. Accurate monitoring of this contaminant is, therefore, essential for ensuring fuel stability and extending engine lifespan. Conventional analytical methods, such as Atomic Absorption Spectroscopy, although highly reliable, demand sophisticated instrumentation and labor-intensive sample preparation. In this study, a sustainable and innovative approach is proposed by combining extraction induced by microemulsion breaking (EIMB) with digital image analysis (DIA) for the determination of iron in gasoline. Exploiting the ethanol naturally present in Brazilian gasoline, microemulsions are formed without the use of surfactants, thereby simplifying the procedure and minimizing reagent consumption. Experimental conditions were optimized through a complete 2^4^ factorial design, leading to the use of isopropanol as a dispersing agent, 1.0 mol L^–1^ HCl, an 8 min extraction time, and a breaking volume of 500 μL. Quantification was performed using smartphone-based DIA, achieving a linear range of 0.3–10 mg L^–1^ and a detection limit of 0.3 mg L^–1^. Recovery tests on spiked gasoline samples confirmed the method’s reliability, with recovery rates ranging from 97.4% to 116.2%. By reducing reagent use, eliminating toxic surfactants, and enabling low-cost, accessible analysis, the proposed EIMB–DIA strategy aligns with Green Chemistry principles and represents a significant advance in sustainable analytical methodologies for complex fuel matrices.

## Graphical abstract

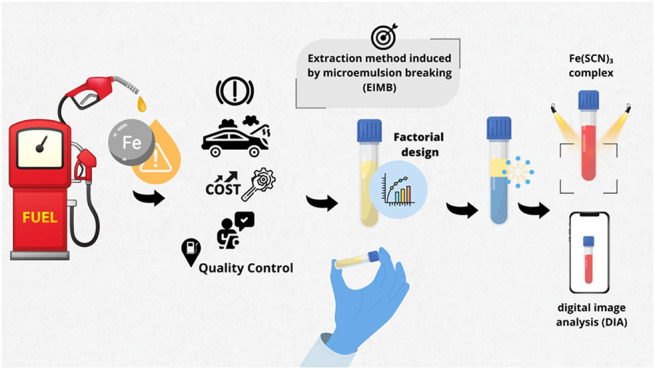

## Introduction

Automotive gasoline mainly contains hydrocarbons and smaller quantities of heteroatomic organic molecules and additives that enhance engine performance.^1,2^ However, due to refining and storage conditions (e.g., time and temperature), gasoline may contain undesirable solid compounds, commonly called gums. The formation of these compounds is attributed to redox reactions between hydrocarbons and atmospheric oxygen, leading to changes in the fuel’s physical and chemical characteristics.^2^ Metals present in gasoline, which may originate from natural petroleum or result from contamination during transport and storage, can catalyze these redox reactions. Components containing lead­(II), manganese­(II), and iron­(III) can damage catalytic converters and cause combustion failures by coating vehicle components such as spark plugs, valves, and cylinders.^3^ In addition to acting as catalysts in gum formation, which reduces engine efficiency, performance, and durability, the presence of metallic ions is also undesirable due to the pollution caused by the products generated during combustion processes.^4,5^ To solve this, the Brazilian National Petroleum Agency (ANP) prohibits the addition of compounds containing metals (e.g., Fe (III)) to automotive gasoline.^6^

The determination of metal ions in gasoline is influenced by the matrix complexity, such as its variable chemical composition, organic contaminants, and low concentration of metals, which often require highly sensitive techniques.^7^ Additionally, gasoline’s physical properties, such as volatility and immiscibility with aqueous solutions, necessitate the use of specific solvents and extraction methods to isolate the analytes effectively. Sample preparation is particularly challenging, usually requiring selective extraction processes to minimize matrix effects and enable accurate quantification. Extraction techniques are an alternative solution to these challenges^8,9^ offering advantages such as eliminating or reducing matrix effects and improving the sensitivity and detection limits of the applied techniques.^10,11^ Recently, a new extraction method induced by microemulsion breaking (EIMB) was developed, in which microemulsions are formed directly in a gasoline sample without the need for the addition of surfactants, due to the high concentrations of ethanol in the Brazilian samples, which facilitates and promotes microemulsion formation.^12,13^ In the case that the samples do not contain ethanol, a small amount of surfactant can be added during the extraction process. Thus, EIMB offers the advantage of facilitating the formation of microemulsions, which are formed instantly without the need for shaking the sample, and also simplifies the breaking of the microemulsion by adding small volumes of aqueous solution, eliminating the need for centrifugation or heating steps.^11,12,14^

Analytical techniques frequently used to monitor iron­(III) ions and other metallic species in fuels are atomic absorption spectrometry (AAS),^12–16^ inductively coupled plasma optical emission spectrometry (ICP-OES),^17–19^ inductively coupled plasma mass spectrometry (ICP-MS),^4,20^ and X-ray fluorescence.^9,21^ Although these techniques offer excellent analytical parameters, such as sensitivity, selectivity, and precision, they demand significant investment for acquisition, occupy large physical space, do not allow on-site analysis, and involve laborious and time-consuming sample preparation steps. AAS is valued for its high sensitivity and specificity, but it is limited to single-element analysis and often requires extensive sample preparation. On the other hand, ICP-OES provides multielement detection capabilities with excellent precision; however, the high operational costs and complex instrumentation limit its routine application. Both techniques demand significant sample preparation efforts, including solvent extraction, matrix elimination, and preconcentration steps, to ensure accurate and reliable quantification in gasoline matrices.^10,22^ These steps, while essential, are time-consuming, increase reagent consumption, and require adherence to strict protocols to avoid contamination and analytical errors.^7,10^

Colorimetric reactions using specific reagents combined with digital image analysis (DIA) emerge as a viable alternative to conventional techniques and have been widely used for the development of alternative quantification methods.^13,14,23^ DIA is an analytical technique based on the acquisition of a digital image—using e.g., scanners, webcams, or smartphones—of the colorimetric reaction, followed by the extraction of color information using a suitable color space model, such as RGB, HSV, or CMYK.^23–25^ The individual values of each channel or different combinations thereof are converted into a suitable analytical signal and correlated with analyte concentration. Capturing images under constant lighting and distance conditions results in increased repeatability, sensitivity, and precision.^23,24^ DIA allows for rapid analysis while reducing reliance on sophisticated instrumentation and reagents.^25^ The advantages of DIA include low cost, simplicity, portability, and adherence to the principles of green chemistry.^26^

Several analytical methods have been reported for the determination of metals using DIA. DIA minimizes reagent consumption and shortens the analysis time, leading to lower operational costs and a reduced environmental footprint compared to traditional methods, as already mentioned. The applications include the detection of Cu­(II) in Brazilian sugarcane spirits,^27^ speciation of iron­(II/III) in white wine,^28^ determination of iron in food,^29^ analyses in green tea^30^ and water samples,^31^ colorimetric detection of mercury Hg­(II),^32^ quantification of copper­(II) and zinc­(II) in urine samples,^33^ and colorimetric analysis of Ni­(II) and Cr­(VI).^34^

This work proposes a new method for determining iron in Brazilian gasoline samples. It combines the extraction technique induced by microemulsion breakdown with colorimetric determination and digital image analysis. This integrated approach leverages the strengths of both techniques and offers a promising solution for iron analysis by reducing analytical complexity and cost.

## Methodology

### Reagents, Solutions, and Samples

All of the reagents were of analytical-grade purity. Ultrapure water with a resistivity of 18.2 MΩ cm was obtained from a purification system (Milli-Q system, Millipore, Bedford, MA, USA). The working solutions were prepared directly in gasoline, containing Fe­(III) ions at a concentration of 0.50 mg L^–1^ by diluting the standard 1000 mg L^–1^ solution (SpecSol, Brazil). The concentrated acids, hydrochloric acid (HCl) and nitric acid (HNO_3_), used in the preparation of the solutions, were of analytical grade (Synth, Brazil). Glassware and the bottles used for preparing and/or storing the solutions were decontaminated in a 10% v/v HNO_3_ bath for 24 h, then washed with deionized water and air-dried at room temperature. In the EIMB procedure, microemulsions were prepared in 15 mL plastic tubes and stirred using a vortex (IKA, Sweden).

For the DIA procedure, the iron­(III) thiocyanate complex (Fe­(SCN)_3_), which is red in color, was prepared by mixing 250 μL of Fe­(III) solution at different concentrations (from 0.25 to 10 mg L^–1^) with 250 μL of 3.50 mol L^–1^ HCl and 150 μL of 1.0 mol L^–1^ NaSCN, resulting in a final sample volume of approximately 0.65 mL. The amount and concentration of HCl used were sufficient to maintain an acidic medium with pH levels between 1 and 2, allowing for the maximum color intensity of the Fe­(SCN)_3_ complex, ensuring the highest analytical response.

### Instrumentation

For the optimization of the EIMB procedure, a flame atomic absorption spectrometer (FAAS, PerkinElmer AAnalyst 400), equipped with a hollow cathode lamp, was employed. The flame was generated from a mixture of acetylene and compressed air. Iron ions were detected at 248.30 nm.

The digital image acquisition chamber was developed based on the device described elsewhere^15^ and consists of a simple MDF (Medium Density Fiberboard) box with dimensions of 15 cm × 10 cm × 6 cm, finished internally with glossy white spray paint. Four white LED lights with a voltage of 12 V, power of 12 W/m, and an emission color of 4000 K were positioned in the internal lid, providing constant and homogeneous lighting for image capturing.

During image capture, the smartphone camera was positioned perpendicularly (90°) to the cuvette at a fixed distance of 6 cm through a frontal aperture that ensured reproducible alignment. The smartphone was externally supported and kept immobile during all acquisitions. The box lid, containing the LED lighting system on its inner borders, was fully closed during image capture to eliminate external light interference. The internal glossy white coating acted as a natural diffuser, providing a uniform light distribution without additional diffusing materials.

### Procedure for the Extraction Optimization

4.6 mL of the gasoline sample containing Fe­(III) ion, 50 μL of acid, and 350 μL of dispersant agent were added to a 15 mL plastic tube and mixed. The tube was sealed, and the microemulsion was obtained after vortex shaking. Subsequently, 400 μL of an acidic solution was added to induce the breakdown of the microemulsion, which occurred instantly. After the breakdown of the microemulsion, two phases were visible: an aqueous phase, composed of acid, water, ethanol (present in gasoline), dispersant agent, and the extracted analytes, and an organic phase, which contained the remaining gasoline. The aqueous phase was collected and analyzed by FAAS.

### Steps for Optimizing the EIMB Procedure

In this experiment, 4.6 mL of a gasoline sample spiked with Fe­(III) at a concentration of 0.5 mg L^–1^ was mixed with 50 μL of HNO_3_ (3.5 mol L^–1^) and 350 μL of the dispersing agent. After the formation of the microemulsion, the mixture was stirred in a vortex for 5 min. Then, 400 μL of HNO_3_ (3.5 mol L^–1^) was added to trigger the breakdown of the microemulsion. The experiments were carried out in triplicate, and the aqueous phase was analyzed using FAAS. After the appropriate dispersing agent was selected, the other variables that influence the extraction procedure were identified and evaluated through a full 2^4^ factorial design. The type of acid (Var 1), acid concentration (mol L^–1^) (Var 2), extraction time (min) (Var 3), and microemulsion breakdown volume (μL) (Var 4) were evaluated. Table 1 lists the values adopted for each experiment.

**Table 1:** 2^4^ Factorial Design for Optimization of the EIMB Procedure

**Run**	**Var 1**	**Var 2**	**Var 3**	**Var 4**
1	(−1) HNO_3_	(−1) 1	(−1) 1	(−1) 100
2	(+1) HCl	(−1) 1	(−1) 1	(−1) 100
3	(−1) HNO_3_	(+1) 3.5	(−1) 1	(−1) 100
4	(+1) HCl	(+1) 3.5	(−1) 1	(−1) 100
5	(−1) HNO_3_	(−1) 1	(+1) 8	(−1) 100
6	(+1) HCl	(−1) 1	(+1) 8	(−1) 100
7	(−1) HNO_3_	(+1) 3.5	(+1) 8	(−1) 100
8	(+1) HCl	(+1) 3.5	(+1) 8	(−1) 100
9	(−1) HNO_3_	(−1) 1	(−1) 1	(+1) 500
10	(+1) HCl	(−1) 1	(−1) 1	(+1) 500
11	(−1) HNO_3_	(+1) 3.5	(−1) 1	(+1) 500
12	(+1) HCl	(+1) 3.5	(−1) 1	(+1) 500
13	(−1) HNO_3_	(−1) 1	(+1) 8	(+1) 500
14	(+1) HCl	(−1) 1	(+1) 8	(+1) 500
15	(−1) HNO_3_	(+1) 3.5	(+1) 8	(+1) 500
16	(+1) HCl	(+1) 3.5	(+1) 8	(+1) 500

### Procedure for the Acquisition of Digital Images

Digital images were captured using the main camera of a Xiaomi Mi A2 smartphone with a resolution of 20 megapixels. The camera was operated in automatic mode. Before each capture, the user tapped the center of the screen to focus on the cuvettes, ensuring consistent focus across all images. No adjustments to exposure, ISO, or white balance were applied.

For this, the samples were placed in glass cuvettes, and the images were captured through the front aperture of the apparatus. The images were then transferred to a computer via USB and analyzed using ImageJ software (version 1.53t), developed by Wayne Rasband at the National Institutes of Health, USA, on the Java platform (version 1.8.0_345).^20^

Regions of interest (ROIs) in ImageJ software were manually selected, prioritizing homogeneous areas devoid of possible shadows and reflections. Preliminary tests indicated that the central region of the image exhibited the highest color uniformity; therefore, the ROIs were selected from this area for all samples. The “Analyze – Histogram” command was employed to obtain the mean intensity values of the RGB channels in the selected ROIs of each sample. The RGB intensity values thus obtained were then converted into a suitable analytical signal (AS) using eq 1

1 $$\mathrm{AS}=-\frac{\log I_{n}}{I_{analytical blank}}$$

In the above equation, *I_n_* represents the average intensity of the R, G, or B channel of the sample, and *I*_analytical blank_ represents the average intensity of the white R, G, or B channel for the analytical blank. After the SA values were obtained the analytical curves were constructed.

### Protocol for the Determination of Iron­(III) Using EIMB-DIA

For the determination of Fe­(III) in gasoline samples, EIMB-DIA was employed: 4.6 mL of gasoline spiked with Fe­(III) was combined with 50 μL of a 3.5 mol L^–1^ acid solution and 350 μL of 1-propanol. The mixture was vortexed for 5 min to ensure homogenization, and subsequently, 400 μL of the same acid solution was added to break the microemulsion. The aqueous phase was then collected and subjected to a colorimetric reaction by mixing 250 μL of this extract with 250 μL of 3.5 mol L^–1^ HCl and 150 μL of 1.0 mol L^–1^ NaSCN, resulting in the formation of a red Fe­(III)-thiocyanate complex.

## Results and Discussion

### Optimization of the EIMB Procedure

Typically, extraction procedures based on the formation of microemulsions require the addition of a dispersing agent. Short-chain alcohols, such as 1-propanol (propyl alcohol), ethanol (ethyl alcohol), 2-propanol (isopropyl alcohol), and 1-butanol (butyl alcohol), were evaluated. Although the composition of Brazilian gasoline contains a reasonable proportion of ethanol (25–27% v/v), a microemulsion for extracting analytes cannot be formed using only a gasoline sample and the addition of the extracting acid solution.^14^ Therefore, the use of dispersing agents remains essential. Dispersing agents are low molecular weight molecules or polymer chains that are typically employed in emulsions or microemulsions to enhance the stability of dispersion.^11^ Herein, 1-propanol (propyl alcohol), ethanol (ethyl alcohol), 2-propanol (isopropyl alcohol), and 1-butanol (butyl alcohol) were evaluated.

The extraction results were evaluated using the relative absorbance (ABSR), calculated according to eq 2

2 $$\mathrm{ABSR}=\frac{A_{\mathrm{experimental}}}{A_{\mathrm{highest}}}$$

where *A*_experimental_ corresponds to the absorbance obtained in each assay, while *A*_highest_ represents the highest absorbance observed among all experiments.

Van der Waals forces, electrostatic interactions, and hydrophobicity must be considered in selecting the most appropriate dispersing agent. Although van der Waals forces are related to dipole interactions, they also involve dispersion and induction components; therefore, they do not depend solely on the dipole moment but on the molecular polarizability and temporary charge fluctuations. Particles in an aqueous medium can agglomerate when the van der Waals attraction exceeds electrostatic repulsion. In response to the addition of suitable dispersants, steric hindrance and electrostatic stabilization occur, preventing agglomeration.

1-Propanol has been widely used as a dispersing agent in the preparation of gasoline microemulsions. Considering the relative absorbance as the analytical signal, the use of isopropanol provided 100% of the analytical signal, while the use of 1-butanol yielded approximately 83%. Ethanol was the least efficient dispersing agent, showing approximately 37% of the analytical signal. Furthermore, microemulsions were readily formed upon using isopropanol.

After the best dispersing agent was selected, the influence of other factors that may affect the EIMB process was studied using a 2^4^ factorial design. All data obtained were processed to determine whether the effects are significant. One of the mechanisms used for this purpose is to create a graphical representation of the probability, along with the percentage of effects. These results are shown in Figures 1A,B.

**Figure 1:**
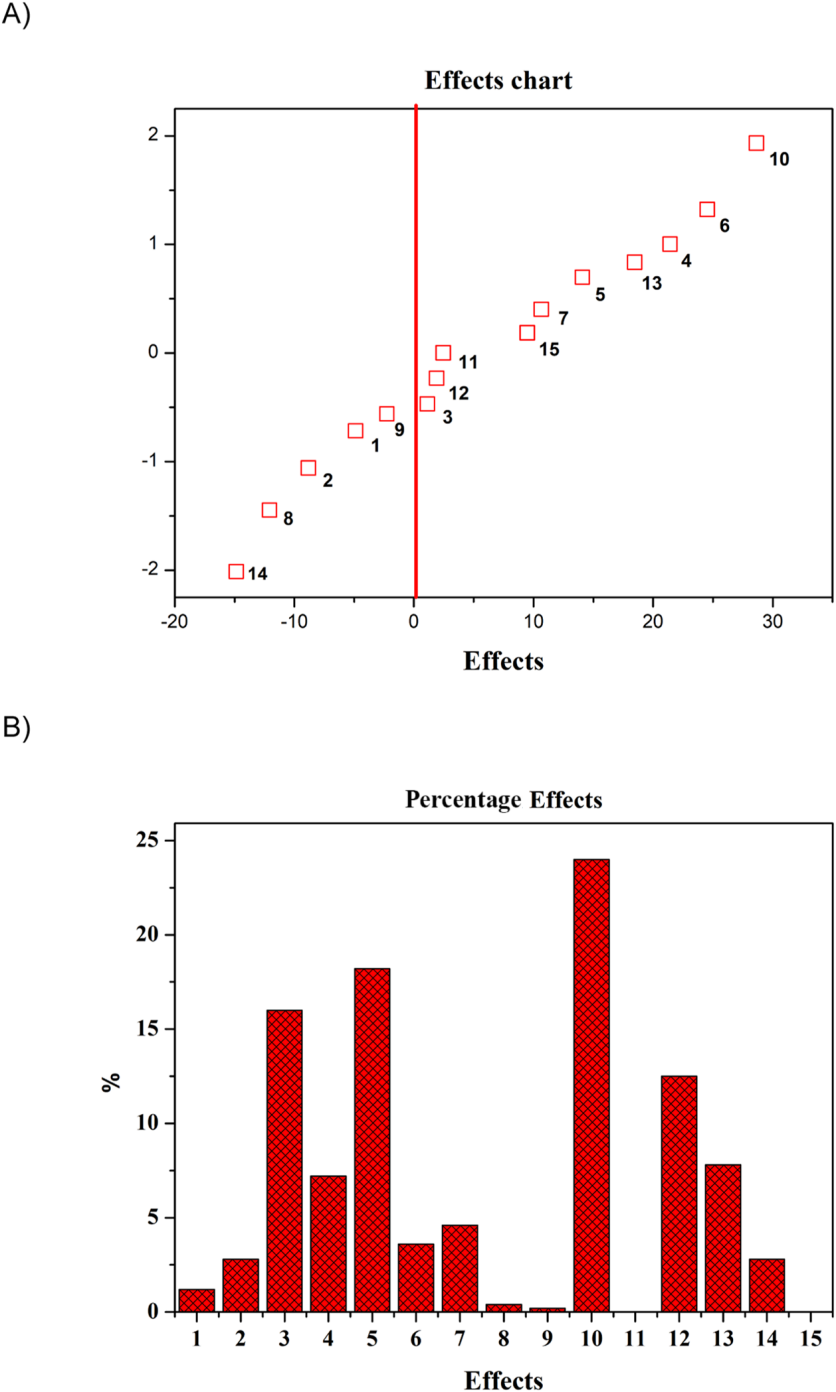
A) Probability of effects in the 2^4^ factorial design of Fe­(III) ion extraction, where the *y*-axis represents the standard normal distribution of the effects, and the *x*-axis displays the estimated effects. B) Percentage of effects for 2^4^ factorial designs. The values obtained for each effect, their percentages, and the respective interaction of each variable corresponding to the effect are found in Table 2.

In the graph of the probability of effects, the ones considered minor are clustered close to the solid red line around zero, while the more significant effects are farther from zero and should be considered when optimizing the procedure. The graph of the percentage of effects illustrates the contribution of each effect to the sum of the squares of the effects. Table 2 presents the values obtained for each evaluated effect, as well as the corresponding interactions between variables. Effect 4, which corresponds to the main effect of variable 4 (breakdown volume), showed a positive value of 22.00 and a percentage of 15.94%. The positive value indicates that the extraction system exhibits a greater analytical response when the variable is maintained at its high level. Therefore, this variable was kept at its maximum level of 500 μL. Effect 10, representing the interactions between variables 3 (extraction time) and 4 (breakdown volume), showed a positive value of 26.75 and a percentage of 23.56%. The positive effect suggests that a stronger analytical response is obtained when both variables are set at the same high or low level. Therefore, considering that the second-greatest effect observed is due to variable 4, it was set at its high level, with variable 3 also set at its high level of 8 min. Approximately 72.87% of the acquired data were evaluated by accounting for important effects. Finally, the optimized conditions for EIMB were defined as isopropanol as a dispersant agent, HCl 1.0 mol L^–1^, extraction time of 8 min, and microemulsion breakdown volume 500 μL.

**Table 2:** Contribution Values of the Effects and Their Corresponding Percentages for Each Evaluated Effect

Effect	Variable or Interaction between variables	Effect contribution	Effect percentage (%)
**1**	Variable 1	–5.75	1.09
**2**	Variable 2	–9.00	2.67
**3**	Variable 3	0.75	0.02
**4**	Variable 4	22.00	15.94
**5**	Variables 1 and 2	14.75	7.16
**6**	Variables 1 and 3	23.50	18.18
**7**	Variables 1 and 4	10.25	3.46
**8**	Variables 2 and 3	–11.75	4.55
**9**	Variables 2 and 4	–3.50	0.40
**10**	Variables 3 and 4	26.75	23.56
**11**	Variables 1, 2, and 3	1.50	0.07
**12**	Variables 1, 2, and 4	1.25	0.05
**13**	Variables 1, 3, and 4	19.50	12.52
**14**	Variables 2, 3, and 4	–15.25	7.66
**15**	Variables 1, 2, 3, and 4	9.00	2.67

### Relation Between Analyte Concentration and Color Intensity

The behavior of RGB color space parameters against different analyte concentrations influences key analytical factors, such as the linear range and linearity. This behavior depends on the color of the solution and the illumination conditions during image acquisition. For instance, excessive illumination can reduce the camera’s ability to detect slight changes of colored surfaces, leading to lower sensitivity. Therefore, selecting the appropriate color channel under optimal illumination conditions is essential for enhancing the analytical performance of a DIA-based method.

To investigate this, the relationship between RGB channels and Fe­(III) concentration after its reaction with thiocyanate was evaluated using different numbers of LEDs positioned on the internal lid of the image acquisition chamber. Iron-thiocyanate complexes with Fe­(III) concentrations ranging from 0.25 to 10.0 mg L^–1^ were prepared and placed in the acquisition chamber, and the digital image was taken. As shown in Figure 2(a–c), both the RGB channel and the number of LEDs influenced the linearity of the response. Among the three channels, the green (G) channel exhibited the highest sensitivity and linearity in detecting changes in the complex concentration, regardless of illumination conditions. This can be attributed to the red color of the complex, which contrasts with the green channel’s complementary response. Additionally, images captured with four LEDs demonstrated the best linearity, with an *r* ^2^ value of 0.9955. Based on these findings, the green channel with illumination from four LEDs was identified as the optimal configuration for image acquisition by using the proposed method.

**Figure 2:**
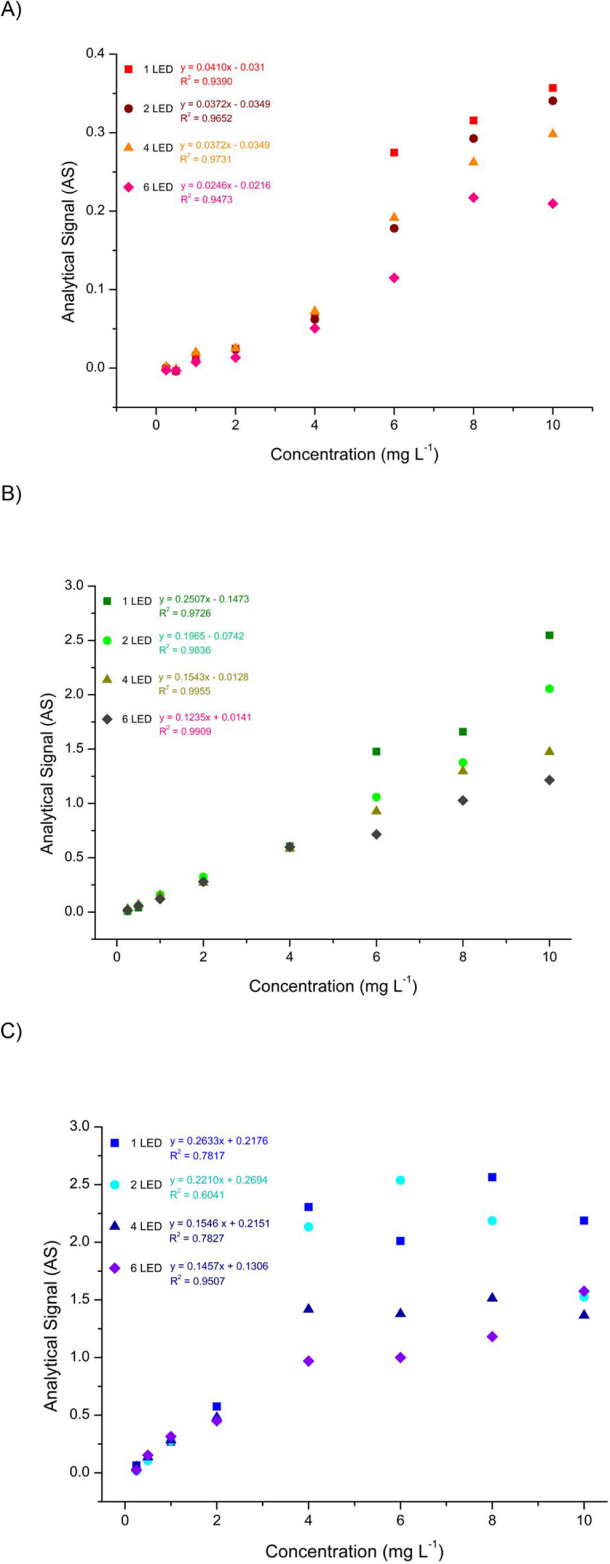
Influence of the number of LEDs used for image acquisition; images were captured using 1, 2, 4, and 6 white LEDs: (A) Red Channel, (B) Green Channel, and (C) Blue Channel.

The use of RGB color space as an analytical signal should also be evaluated by comparing different devices with varying specifications to determine whether a digital image-based methodology is affected by the choice of electronic device. In this context, the behavior of RGB signals was compared using images captured with three smartphones: Xiaomi Mi A2, Apple iPhone XS Max, and Samsung Galaxy A13.

Analytical curves were constructed based on data extracted from the green (G) channel for each device. As shown in Figure 3, the slope, intercept, and correlation coefficient of the G channel response varied depending on the smartphone model. This indicates that the digital images used for measurements must be taken with the same device to ensure accuracy. If different devices are used (e.g., analyses conducted in multiple locations), a new calibration curve must be generated for the specific device employed in the sample analysis. Additionally, the sensitivity varies across smartphone models, as reflected in the differences in the slope values of the analytical curves. Therefore, the smartphone model Xiaomi Mi A2 was used in this study.

**Figure 3:**
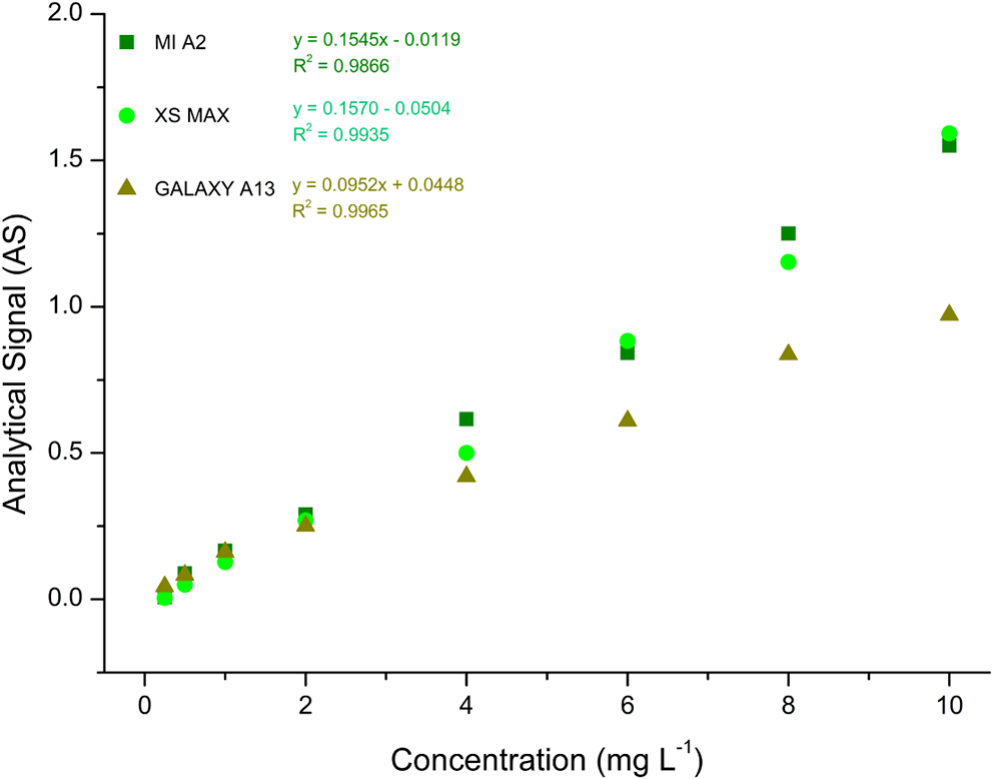
Assessment of the reproducibility of the method using different smartphones.

### Analytical Parameters

After the experimental conditions were optimized, the analytical performance of the proposed method was obtained. First, precision was evaluated by measuring the analytical signal of spiked samples at three concentration levels (0.5, 4, and 8 mg L^–1^). Results were expressed as the relative standard deviation (RSD). Intraday and interday precision (*n* = 3), ranging from 2.7% to 5.0% and 4.1% to 6.2%, respectively, were obtained. A suitable linear response was observed within the 0.25 to 10 mg L^–1^ range, with an *r* ^2^ value of 0.9955. The limit of detection (LOD) was visually determined to be 0.1 mg L^–1^, defined as the lowest concentration at which the signal could be distinguished from the blank. The limit of quantification (LOQ) was estimated as three times the LOD, resulting in 0.3 mg L^–1^. Table 3 summarizes the analytical performance of the proposed method.

**Table 3:** Summary of the Analytical Parameters of the Proposed EIMB-DIA Method for the Determination of Fe­(III)

**ParametersParameters**	**Values**
**Linear range** mg L^**–1**^	0.25–10
***R***	0.9988
***R*** ^**2**^	0.9955
**Intraday repeatability (DPR%, 4.0** mg L^**–1**^, ***n* = 3)**	4.21
**LOD (mg L** ^**–1**^ **)**	0.1
**LOQ (mg L** ^**–1**^ **)**	0.3
**Extraction time**	8 min
**Volume sample (mL)**	4.6

Next, accuracy was evaluated through a recovery test using gasoline samples spiked with known amounts of Fe­(III) and analyzed using the EIMB-DIA method. The Fe­(III) concentration was determined by using the previously established calibration curve, and recovery percentages were calculated by comparing the measured concentration with the spiked concentration. As shown in Table 4, recovery rates ranged from 92.3% to 116.2%, falling within the acceptable range of 80% to 120%. Besides, the analytical performance of the developed EIMB-DIA analytical method was compared with that of similar studies for the determination of iron in a variety of samples (Table 5). The comparative analysis presented emphasizes some analytical parameters of each approach rather than a full analytical validation data set.

**Table 4:** Recovery Test Applied to Gasoline Samples After EIMB Extraction and Detection by DIA^a^

	**Sample 1**		**Sample 2**		**Sample 3**		**Sample 4**	
**Fe(III) concentration**	mg L^–1^	*R* (%)	mg L^–1^	*R* (%)	mg L^–1^	*R* (%)	mg L^–1^	*R* (%)
Not spiked	<LOD	-	<LOD	-	<LOD	-	<LOD	-
0.5 mg L^–1^	0.58	116.2	0.58	115.1	0.56	112.8	0.55	109.7
4.0 mg L^–1^	3.89	97.4	3.97	99.1	3.98	99.5	4.18	104.5
8.0 mg L^–1^	8.24	103.1	7.38	92.3	8.46	105.8	8.83	110.4

a R: Recovery rate (%).

**Table 5:** Comparison of the Analytical Performance of the EIMB-DIA Analytical Method with Studies Published Previously on the Determination of Iron

**Method**	**LOD** **(mg L** ^**–1**^)	**LOQ** **(mg L** ^**–1**^)	**Linear range** **(mg L** ^**–1**^)	**Sample**	**Method for image acquisition**	**Ref.**
EIMB-DIA	0.1	0.3	0.25 −10	gasoline	Smartphone	This work
Dispersive liquid–liquid microextraction (DLLME)	0.014	0.0465	0.047–1.0	water and food	Digital scanning	^35^
Digital image-based flow-batch analyzer (DIB-FBA)	0.002	0.0772	0.1–1.0	tomato	Webcam	^29^
Colorimetric reaction	0.042	0.141	0.25–2	white wine	Microsoft 720p HD video chat webcam	^28^
Colorimetric reaction	0.1	-	0.5–10	bioethanol fuel	Smartphone	^25^
Colorimetric reaction	0.9	1.6	2.5–17.5	green tea (*Camellia sinensis*)	Canon SX620HS digital camera	^30^
Colorimetric reaction	0.1	0.3	10–80	water	Huawei P10 mobile phone equipped	^36^

### Evaluation of Analytical Greenness

The analytical greenness of the proposed EIMB-DIA method was calculated using the AGREEprep tool (Analytical Greenness Metric for Sample Preparation).^26^ This approach considers factors such as sample preparation, reagent and energy consumption, waste generation, and the adoption of more sustainable laboratory practices. As shown in the pictogram in Figure 4, the method received a score of 0.55, indicating that it is partially favorable in minimizing environmental impact and aligns with Green Chemistry principles. The score is influenced by both the use of EIMB as a sample preparation technique and the determination through DIA. The EIMB contributes to a lower volume of hazardous solvents used, requires fewer sample preparation steps, and demands a smaller sample volume for extraction. In turn, the determination of iron ions by DIA enables a detection method with low energy consumption, lower cost, and reduced reagent volume compared to other analytical detection techniques. Additional details regarding the scoring criteria, color coding, and the overall greenness assessment (score = 0.55) are provided (Table S1), which contains the report generated by the AGREEprep platform for the Fe­(III) detection method by DIA after EIMB.

**Figure 4:**
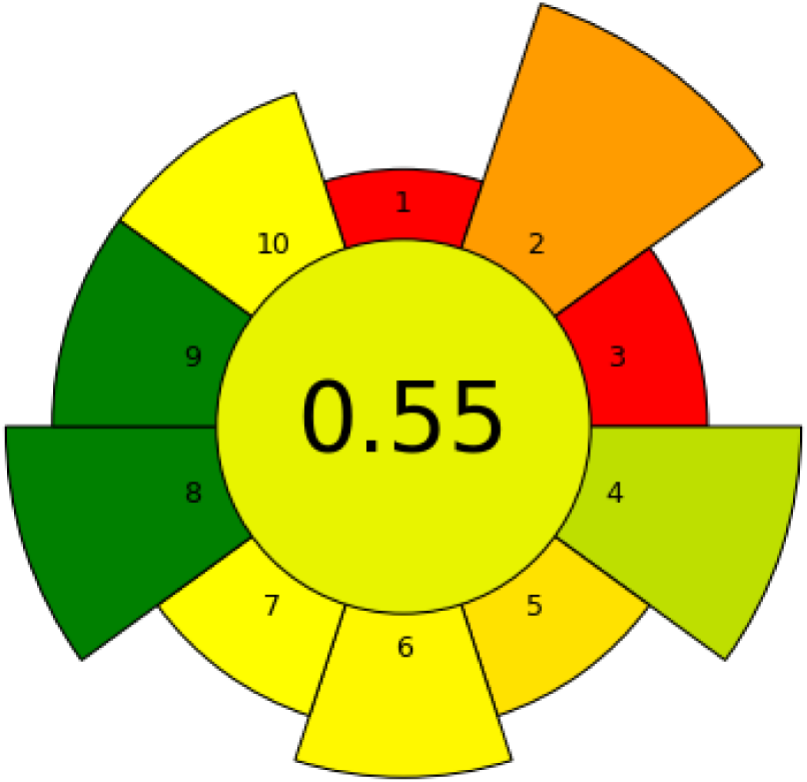
AGREEprep pictogram of Fe­(III) detected in gasoline per DIA.

## Conclusions

The quantification of metal ions, such as Fe­(III), in gasoline samples is typically performed by using bulky and expensive analytical techniques. In this study, we propose a sustainable, low-cost, miniaturized, and yet sensitive analytical method for Fe­(III) determination in gasoline by combining extraction-induced microemulsion breaking (EIMB) with digital image analysis (DIA).

The EIMB extraction method enabled emulsion formation without the need for surfactants, enhancing the sustainability of the approach. A classical colorimetric reaction with SCN^–^ was used for Fe­(III) quantification, producing a red-colored complex. Image acquisition was performed using a smartphone, improving the portability of the analytical method. Although this colorimetric reaction has previously been applied for the determination of Fe­(III), integrating the EIMB method with DIA proved to be an excellent alternative for metal quantification in complex matrices, such as fuel samples. Thus, the proposed method can be readily applied in fuel quality control, aiding in the prevention of fuel adulteration while minimizing risks to consumers and the environment.

## Supplementary Material



## Notes

Table summarizes the report generated by the AGREEprep platform for the Fe­(III) detection method using DIA after EIMB (PDF)
