# Supplementary material for: Green and Efficient Determination of Iron(III) in Gasoline Using Microemulsion-Based Extraction and Digital Image Analysis
Source: ACS Omega. 2025 Dec 3;10(49):60603–11. doi: 10.1021/acsomega.5c08435 (PMC12713479; doi:10.1021/acsomega.5c08435)
Supplement: Supplementary file 1 [file ao5c08435_si_001.pdf]

SUPPLEMENTARY MATERIAL

Green and Efficient Determination of Iron(III) in Gasoline Using Microemulsion-Based Extraction and Digital Image Analysis

Jordana de Assis Nunes Oliveira[a], James Michael Silva[a], Eduarda Garcia Santana[a], Wallace Henrique Cardoso Peres[a], Weida Rodrigue Silva[a], João Flávio da Silveira Petruci[b] and Vanessa Nunes Alves [a]

[a]Federal University of Catalão (UFCAT), Institute of Chemistry, Catalão, GO, Brazil

[b]Federal University of Uberlândia (UFU), Institute of Chemistry, Uberlândia, MG, Brazil

Supplementary Material

Table S1: Report generated by the AGREEprep platform for the Fe(III) detection method by DIA after EIMB.

Number in pictograme	Criterion	Answer	Score	Weight
1	Sample preparation placement	Ex situ	0.00	1
2	Hazardous materials	1.2 mL	0.31	5
3	Sustentainability, renewability and reusability of materials	< 25% of reagentes and materials are susteinable or renewable, but can only be used once	0	2
4	Waste	1 mL	0.63	4
5	Size economy of the sample	Volume of the sample: 4.6 mL	0.45	2
6	Sample throughput	8 samples/h	0.49	3
7	Integration and automatioln	Sample prep. Steps: 2 steps, Semi-automated systems	0.50	2
8	Energy consumption	3.8 [W]	1.00	4
9	Post-sample preparation configuration for analysis	Simple, readily available detection: smartphones	1.00	3
10	Operator’s safety	2 hazards	0.50	3
